# Colchicine and coronary heart disease risks: A meta-analysis of randomized controlled clinical trials

**DOI:** 10.3389/fcvm.2022.947959

**Published:** 2022-09-12

**Authors:** Zijun Ma, Jun Chen, Kaiqin Jin, Xin Chen

**Affiliations:** ^1^Sinopharm Dongfeng General Hospital, Hubei University of Medicine, Shiyan, China; ^2^Department of Cardiology, The Second Affiliated Hospital of Anhui Medical University, Hefei, China

**Keywords:** coronary heart disease, secondary prevention, colchicine, dose, randomized controlled trial

## Abstract

**Background:**

Several trials have considered the safety and clinical benefits of colchicine as a treatment option for secondary prevention in patients with coronary atherosclerotic heart disease (CAD), but its safety and clinical benefits remain controversial. The purpose of this study was to explore the clinical benefits of colchicine, focusing on certain subgroups of patients.

**Methods:**

Randomized controlled trials (RCTs) of colchicine in subjects with acute or chronic CAD compared with controls were included to assess all-cause mortality, non-cardiovascular mortality, gastrointestinal adverse effects, diarrhea, MACE, cardiovascular mortality, MI, stroke, and revascularization. We analyzed the association of cardiovascular, mortality, and gastrointestinal risk with colchicine in all subjects. We also focused on the cardiovascular risk of colchicine in subgroups with different drug doses, different treatment durations, age, gender, and associated comorbidities.

**Results:**

This meta-analysis included 15 clinical RCTs, including 13,539 subjects. Colchicine reduced the risk of MACE (RR: 0.65; 95% CI: 0.38–0.77, p for heterogeneity < 0.01; I2 = 70%; *p* < 0.01), stroke (RR: 0.48; 95% CI: 0.30–0.76; p heterogeneity = 0.52; I2 = 0%; *p* < 0.01), MI by 40% (RR: 0.60; 95% CI: 0.43–0.83; p for heterogeneity = 0.01; I2 = 59%; *p* < 0.01) and risk of revascularization (RR: 0.68; 95% CI: 0.56–0.83; p for heterogeneity = 0.17; I2 = 40%; *p* < 0.01), but had no significant effect on risk of cardiovascular death and risk of all-cause mortality. In addition, colchicine increased the risk of gastrointestinal side effects and diarrhea. In a subgroup analysis, low-dose colchicine and treatment duration > 1 month reduced the risk of MACE, MI, stroke, and revascularization. Also, the cardiovascular benefits of colchicine were observed in subjects up to 65 years of age. The results showed that hypertension and diabetes did not have a specific effect on colchicine and MACE risk.

**Conclusion:**

Colchicine has a positive effect in reducing the incidence of MACE, MI, stroke, and revascularization, but can increase the risk of gastrointestinal and diarrhea events. Low-dose colchicine significantly reduces the risk of MACE more than high-dose colchicine, and the benefits of long-term treatment are higher than those of short-term treatment. Long-term low-dose colchicine treatment may significantly reduce the risk of cardiovascular events. Furthermore, colchicine significantly reduced the risk of cardiovascular events in patients up to 65 years of age, but it did not appear to reduce cardiovascular risk in patients over 65 years of age or in preoperative PCI patients.

**Systematic review registration:**

[https://www.crd.york.ac.uk/prospero/], identifier [CDR42022332170].

## Introduction

In recent years, there has been increasing evidence that inflammation plays a key role in the development of atherosclerosis and other cardiovascular diseases (1, 2). Colchicine is a drug with potent anti-inflammatory effects (3). At low doses, it inhibits microtubule growth, while at high doses it supports microtubule depolymerization. Colchicine’s effect on microtubule protein disruption inhibits the action of the NLRP3 inflammasome, resulting in reduced secretion of pro-inflammatory cytokines and inhibition of neutrophil extracellular traps (NETs) formation (4–6). In this context, colchicine has emerged as a new treatment option for cardiovascular diseases.

Clinical trials on the effects of colchicine on cardiovascular-related outcomes in patients with coronary artery disease continue to emerge, Many clinical studies have shown that colchicine significantly reduces the risk of cardiovascular events in patients with coronary artery disease (7, 8), and Several meta-analyses have also shown that colchicine reduces inflammation levels in patients with unstable coronary atherosclerotic heart disease (CAD) (9) and may be considered as a first-line treatment for secondary prevention in patients with coronary artery disease (10). Few meta-analyses, however, have focused on the long-term cardiovascular risk of colchicine in patients of varied ages, as well as the cardiovascular outcomes of PCI pre-operative treatment. Therefore, we conducted a meta-analysis to evaluate the clinical efficacy and safety of colchicine in the secondary prevention of coronary heart disease, We also focused on the differences in cardiovascular events between studies based on follow-up duration and age, as well as the relationship between colchicine and cardiovascular events in terms of dose, gender, and associated comorbidities.

## Materials and methods

This meta-analysis followed Preferred Reporting Items for Systematic Reviews and Meta-Analysis (PRISMA) guidelines (11). The protocol of this meta-analysis was registered on the PROSPERO database^1^ with Registration Number 42022332170.

### Search strategy

The search strategy was conducted in accordance with the Participant, Intervention, Comparison, Outcome, and Study Design (PICOS) format as follows: P = adults at least 18 years old with CAD or diagnosed CAD; I = Colchicine; C = control group with or without placebo; O = primary outcome was cardiovascular outcomes, including major cardiovascular events (MACEs), coronary revascularization and all-cause death. Secondary outcomes were non-cardiovascular mortality, gastrointestinal adverse events, and diarrhea; MACEs refer to cardiovascular death, myocardial infarction (MI), and non-fatal ischemic stroke. S = Randomized controlled trials (RCT).

We searched databases including PubMed, Cochrane library, and Clinicaltrial.gov to screen all the eligible RCTs published before 2022.4.20, Language is limited to English. The keyword terms used were “colchicine” and “coronary heart disease” or “coronary syndrome” or “myocardial infarction” or “STEMI” or “stable angina” or “PCI” or “percutaneous coronary intervention” and “randomized controlled trial” (see Supplementary Material for detailed database search strategies). Trials were included if they met the following criteria. If multiple reports described the same trial, the most recent full text was selected for inclusion in this study.

#### Inclusion criteria

The RCTs enrolled adults over the age of 18 with coronary artery disease, regardless of whether they had undergone PCI. No restrictions on country/region, language, or race.

The RCT was designed to compare colchicine treatment with a control group with or without a placebo.

The outcomes of the RCT included one of the following events: MACE; cardiovascular death; MI; stroke; and revascularization; all-cause death; non-cardiovascular death; gastrointestinal adverse effects; diarrhea.

### Data extraction

In each RCT, we extracted the first author, publication year, trial location, participant characteristics, a dose of colchicine, treatment duration, subject number of colchicine treatment group and control group, Mean age of subjects, the sex ratio of colchicine treatment and control groups, number of diabetes and non-diabetes, follow-up time, reported endpoints, and study design.

CAD is defined as an acute or chronic coronary syndrome (CCS). (i) Acute coronary syndromes (ACS) include unstable angina, non-ST-segment elevation myocardial infarction (NSTEMI) and ST-segment elevation myocardial infarction (STEMI). (ii) CCS, also called stable angina or stable ischemic heart disease, which includes a history of angina symptoms, asymptomatic myocardial ischemia, or myocardial revascularization in patients with stable angina.

The final results of the included studies were completed independently by the two researchers, and any disagreements were resolved through consultation.

### Assessment of methodological quality

We assessed the risk of bias for inclusion in the methodological quality of RCTs based on the Cochrane Collaboration risk of bias tool (11): Elements of the Cochrane Collaboration risk of bias tool for assessment included random sequence generation, allocation concealment, participant and personnel blinding, blinding of outcome assessment, incomplete outcome data, no selective outcome reporting and other sources of bias. Any disagreements in the quality assessment are resolved through discussion between the two evaluators and, if necessary, the involvement of a third reviewer to reach a consensus.

### Subgroup analysis

Several RCTs showed that the most common side effect of oral colchicine is gastrointestinal discomfort (12, 13). This effect is dose-dependent and can resolve during continued treatment or after withdrawal of colchicine (14). To identify the effect of colchicine dose on acute or chronic CAD, we divided the included studies into low-dose studies with a dose of 0.5 mg and high-dose studies with a dose of 1 mg.

To further analyze the effect of study follow-up time on the outcome endpoints, we performed subgroup analyses according to the length of follow-up in three subgroups: ≤ 1 month, > 1 month and < 1 year, and ≥ 1 year (median follow-up time). In addition, we analyzed a study on the preoperative treatment of PCI with colchicine, A meta-regression analysis of age was also conducted to determine the correlation between the variables and the results, we performed meta-regression analyses of age to find correlations between variables and outcomes, we also conducted subgroup analyses of age (mean age), sex, and associated comorbidities to find out the factors influencing colchicine on cardiovascular outcomes.

### Statistical analysis

We analyzed the number of endpoint events and the number of patients in the included RCT and subgroup data. We assessed the risk of bias using Peter’s test and regression test for funnel plot asymmetry. I2 and *p-*values were used to test for heterogeneity in each RCT. A fixed effects model was used when I2 < 50% and *P* > 0.10. A random-effects model was used if I2 > 50% or *P* < 0.10. We performed sensitivity analyses to reduce and exclude sources of heterogeneity: (1) When at least three RCTs were combined for the same endpoint outcome, we removed each study in turn and measured the change in I2. If omitting a particular RCT resulted in a significant decrease in I2, that RCT was the cause of heterogeneity. (2) The meta-regression method was used to investigate the relationship between subject age, nationality, and outcome. We performed subgroup analyses according to colchicine dose, study follow-up time, age, the timing of dosing, smoking, hypertension, diabetes or not, and gender. In this meta-analysis, *p* < 0.05 was considered statistically significant. R (version 4.1.2) was used to calculate statistical tests [relative risk (RR), confidence intervals, sensitivity analysis, and I2 tests]. Tables, regression plots, and forest plots generated by R (version 4.1.2) were used to display the data.

## Results

**FIGURE 1:**
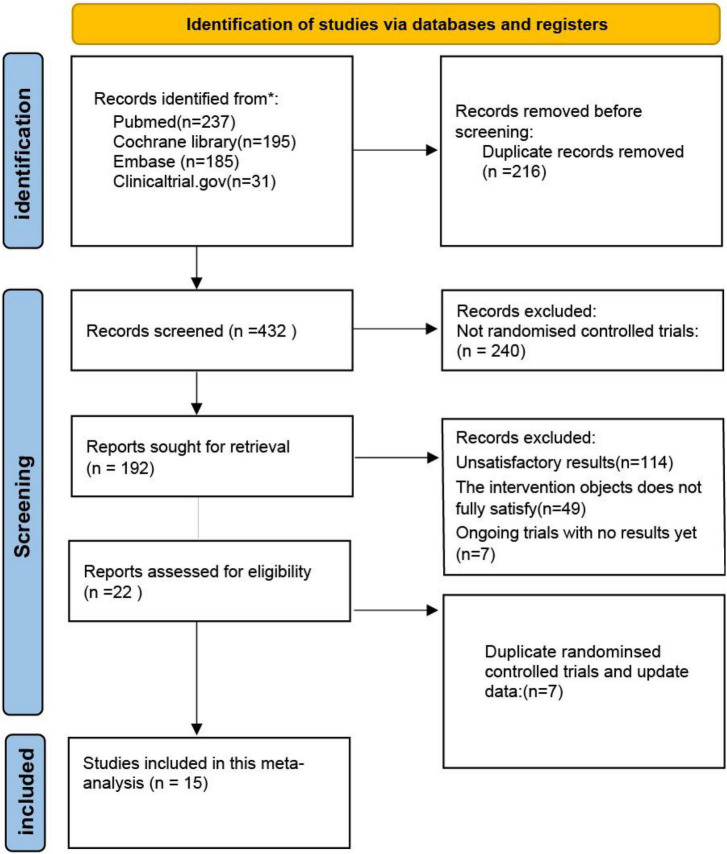
Flow diagram of the study selection process.

This study retrieved 648 articles, and 432 studies were identified after eliminating duplicates. Subsequently, after excluding non-RCTs, intervention subjects, outcome indicators that did not match, and ongoing clinical trials with preliminary results, we included 15 RCTs in our meta-analysis. including 13,543 subjects(Figure 1). These subjects included patients with both acute and CCS, and a proportion of the population had undergone PCI. A total of 6,817 subjects were treated with colchicine, whereas 6,726 subjects were in the control trial. Figure 1 displays the determination of relevant RCTs and finally retrieved the process of obtaining the final literature. Table 1 shows the characteristics of the finally included 15 RCTs (see Supplementary Material).

**TABLE 1:** Main characteristics of included RCTs.

Study	Country	Study design	Character istics	Participants, *n* (%) (treatment/control)		Mean age (years) (treatment/control)		Male (treatment/control)		Diabetes mellitus, *n* (%) (treatment/control)		Dose	Endpoints assessed	Follow-up duration (months)
O’Keefe et al. (24)	America	Single center, double-blind RCTs	CCS patients undergoing angioplasty	130	67		62	111	58	16	8	0.5 mg twice daily	1. Mean coronary artery lumen diameter 2. Recurrent ischemia 3. Adverse reactions 4. All-cause death	5.5
Raju et al. (17)	Australia	Single center, double-blind RCTs	ACS patients	40	40	59	57.2	34 (85%)	37 (92.5%)	7 (17.5%)	6 (15%)	1 mg per day	1. The blood level of hs-CRP 2. platelet function 3. Death 4. myocardial infarction 5. stroke 6. Adverse events	1.03
Nidorf et al. (13)	Australia	Single center, triple-blind RCTs	Stable coronary disease	282	250	66 ± 9.6	67 ± 9.2	222 (89%)	251 (89%)	69 (28%)	92 (33%)	0.5 mg per day	1. Acute coronary syndrome 2. Out-of-hospital cardiac arrest 3. Non-cardiac ischemic stroke 4. Death	36
Deftereos et al. (25)	Greece	Single center, double-blind RCTs	Diabetic ACS and CCS patients undergoing PCI with BMS	100	96	63.6 ± 6.9	63.7 ± 7.2	63 (63%)	65 (68%)	100 (100%)	96 (100%)	0.5 mg twice daily	1. Angio-ISR 2, IVUS-ISR 3, angiographic and IVUS parameters of lumen loss and in-stent neointimal hyperplasia 4. Death events 5. Coronary revascularization 6. Adverse reactions	6
Gianno poulos et al. (18)	Greece	Single center, triple-blind RCTs	ACS and CCS patients undergoing CABG	30	29	64.9 ± 10.1	65.6 ± 9.5	21 (70%)	20 (69%)	11 (38%)	14 (47%)	0.6 mg twice daily	1. Maximal hsTnT concentration within 48 h after surgery 2. Maximal CK-MB levels and area 3. Adverse reactions	8 days after surgery.
Deftereos et al. (19)	Greece	Single center, triple-blind RCTs	ACS patients	77	74	58	58	52 (68%)	52 (70%)	19 (26%)	13 (17%)	0.5 mg twice daily	1. CK-MB 2. hs-TnT 3. Left ventricular ejection fraction 4. Adverse reactions 5. Death events	Lasting 5 days
Zarpelon et al. (20)	Brazil	Single center, double-blind RCTs	ACS and CCS patients undergoing AF-POMR	71	69	61.5 ± 10.3	60.3 ± 8.1	49 (69%)	46 (66.7%)	42 (59.2%)	30 (43.5%)	0.5mg twice daily	1, AF-POMR rate 2, death from any cause 3, hospital length of stay 4, postoperative infection.	Hospitali zation time
Akodad et al. (15)	France	Single center, double-blind RCTs	ACS patients undergoing PCI	23	21	60.1 ± 13.1	59.7 ± 11.4	19 (82.5%)	16 (76.2%)	3 (13%)	3 (14.3%)	1 mg per day	1, CRP peak value during the index hospitalization 2, troponin peak 3, tolerance of colchicine 4, hospitalization duration, 5, major adverse cardiac events	1
Hennessy et al. (23)	Australia	Single center, double-blind RCTs	ACS patients	119	118	61	61	89 (75%)	93 (79%)	27 (23%)	25 (21%)	0.5 mg per day	1. The proportion of patients with a residual CRP level ≥ 2 mg/L at 30 days 2. 30-day CRP changes 3. The proportion of recruited patients completing the study; 4. Adverse events; 5. Participant-reported compliance with study medications; 6. Death and major cardiovascular events	1
Mewton et al. (26)	Iran	Single center, double-blind RCTs	ACS patients undergoing PCI	101	91	NC	NC	NC	NC	NC	NC	NC	1, Thrombolysis in myocardial infarction (TIMI) score; 2, TMPG; 3, TFC; 4, MACE	1
Tardif et al. (8)	Canada	Multicenter, triple-blind RCTs	ACS and CCS patients undergoing PCI	2,366	2,379	60.6 ± 10.7	60.5 ± 10.6	1,894 (80.1%)	1,942 (81.6%)	462 (19.5%)	497 (20.9%)	0.5 mg per day	1. The proportion of patients with a residual CRP level ≥ 2 mg/L at 30 days 2. 30 days CRP change 3. the proportion of recruited patients completing the study; 4. adverse events; 5. participant-reported compliance with study medications; 6. death and major cardiovascular events	22.6
Tong et al. (22)	Australia	Multicenter, triple-blind RCTs	ACS or CCS patients	396	399	59.7 ± 10.2	60.0 ± 10.4	322 (81%)	310 (78%)	75 (19%)	76 (19%)	0.5 mg per day	A residual CRP level ≥ 2 mg/L at 30 days 2. 30 days CRP change 3. the proportion of recruited patients completing	12
Shah et al. (16)	Germany	Single center, triple-blind RCTs	ACS or suspected ischemic heart disease patients with possible PCI	206	194	65.9 ± 9.9	66.6 ± 10.2	193 (93.7%)	181 (93.3%)	114 (55.3%)	117 (60.3%)	1.8 mg before under went PCI	The study; 4. adverse events; 5. participant-reported	1
Nidorf et al. (7)	Australia	Multicenter, triple-blind RCTs	ACS or CCS patients	2,762	2,760	65.8 ± 8.4	65.9 ± 8.7	2,305 (83.5%)	2,371 (85.9%)	492 (17.8%)	515 (18.7%)	0.5mg per day	Compliance with study medications; 6. death and	28.6
Akrami et al. (12)	Iran	Single center, triple-blind RCTs	ACS patients undergoing PCI or medical treatment	120	129	56.9 ± 7.56	56.89 ± 7.45	86 (71.7%)	87 (67.4%)	27 (22.5%)	32 (24.8%)	0.5 mg per day	Major cardiovascular events	6

**FIGURE 2:**
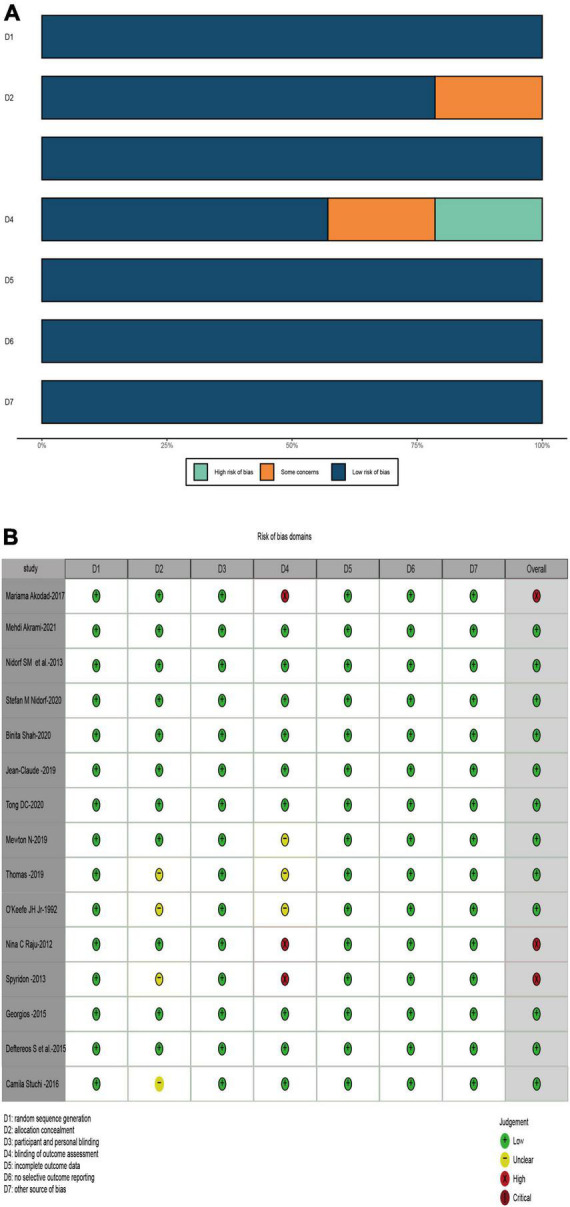
Risk of bias plot. **(A)** Risk of bias summary; **(B)** risks of bias of each included study.

According to the design of each RCT, we used the Cochrane tool to score 15 RCTs for risk of bias. Figure 2 demonstrates the methodological quality for each RCT and showed the risk of bias of RCTs included in our meta-analysis was low (Figure 2).

### Endpoints

#### Cardiovascular outcomes

**FIGURE 3:**
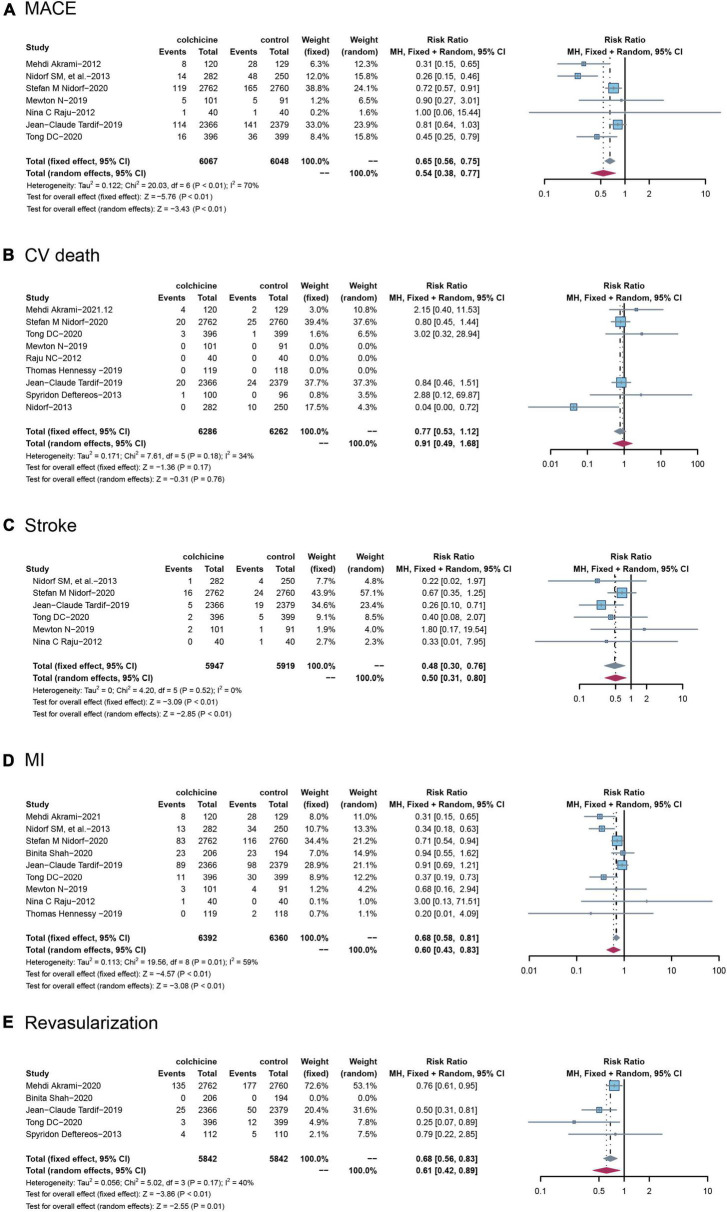
Comparison of colchicine treatment vs. control group on the risks of **(A)** MACE, **(B)** CV death, **(C)** stroke, **(D)** MI, **(E)** Revascularization. MACE, major adverse cardiovascular events; CV death, cardiovascular death; MI, myocardial infarction.

Among the included RCTs, a total of seven studies reported MACE, defined as a composite of cardiovascular death, non-fatal ischemic stroke, and non-fatal MI), with nine RCTs reporting cardiovascular death, six RCTs reporting stroke, and nine RCTs reporting MI, and five RCTs reporting revascularization, respectively. Compared with controls, treatment with colchicine reduced the risk of MACE by 46% (RR: 0.65; 95% CI: 0.38–0.77, *p* for heterogeneity < 0.01; I2 = 70%; *p* < 0.01) and stroke by 52% (RR: 0.48; 95% CI: 0.30–0.76; p for heterogeneity = 0.52; I2 = 0%; *p* < 0.01), a 40% reduction in risk of MI (RR: 0.60; 95% CI: 0.43–0.83; p for heterogeneity = 0.01; I2 = 59%; *p* < 0.01), a 32% reduction in risk of incidence of revascularization (RR: 0.68; 95% CI: 0.56–0.83; p for heterogeneity = 0.17; I2 = 40%; *p* < 0.01). However, colchicine did not reduce the risk of cardiovascular death compared with controls(RR: 0.77; 95% CI: 0.53–1.12; p for heterogeneity = 0.18; I2 = 34%; *p* = 0.17) (Figure 3).

#### All-cause and non-cardiovascular deaths

**FIGURE 4:**
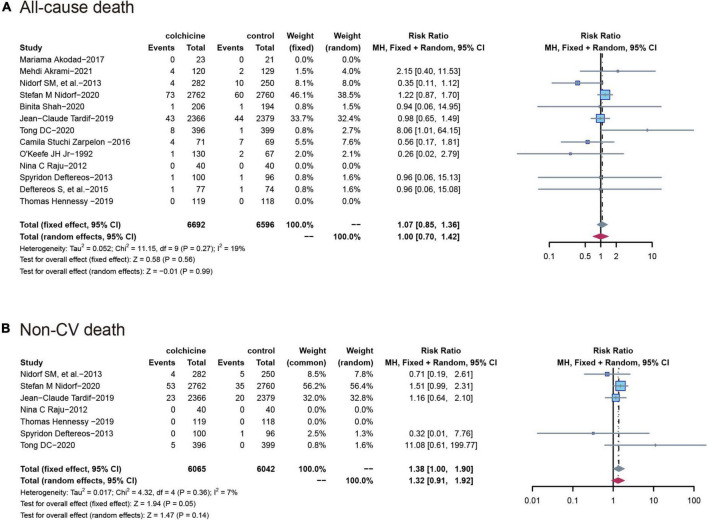
Comparison of colchicine treatment vs. control group on the risks of **(A)** All-cause death; **(B)** non-CV death. Non-CV death, Non-cardiovascular death.

All-cause mortality was reported in 13 trials (*n* = 13,288) and colchicine did not reduce the risk of death from any cause compared with controls (RR: 1.07; 95% CI: 0.85–1.36; p for heterogeneity *p* = 0.56; I2 = 19%; *p* = 0.27), Seven studies reported non-cardiovascular mortality, and similarly, colchicine was not significantly associated with the risk of non-cardiovascular mortality compared with controls (RR: 1.38; 95% CI: 1.00–1.90; p for heterogeneity *p* = 0.36; I2 = 7%; *p* = 0.05) (Figure 4).

#### Gastrointestinal adverse events and diarrhea

**FIGURE 5:**
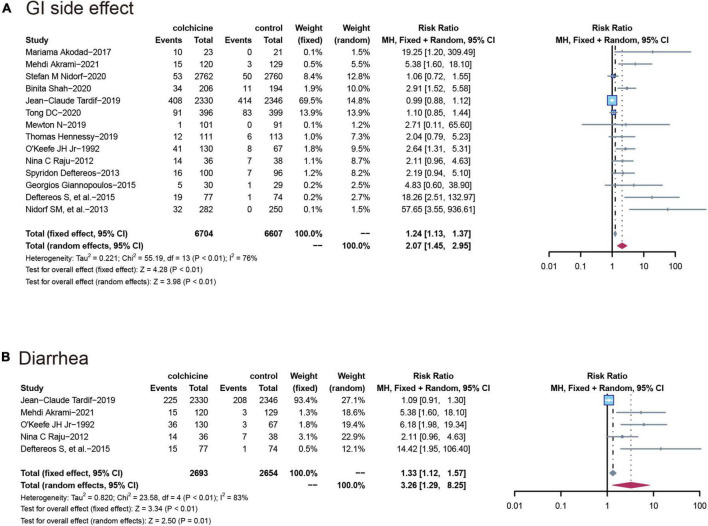
Comparison of colchicine treatment vs. control group on the risks of **(A)** GI side effect; **(B)** diarrhea. GI side effect, gastrointestinal side effects.

All 14 RCT (*n* = 13,311) reported gastrointestinal adverse events, with a significantly higher incidence in the colchicine treatment group than in the control group (RR: 2.07; 95% CI: 1.45–2.95; *p* for heterogeneity *p* < 0.01; I2 = 76%; *p* = 0.04). Also, the risk of diarrhea was higher in the colchicine treated group compared to the control group (RR: 3.26; 95% CI: 1.29–8.25; p for heterogeneity *p* < 0.01; I2 = 83%; *p* = 0.01) (Figure 5).

**FIGURE 6:**
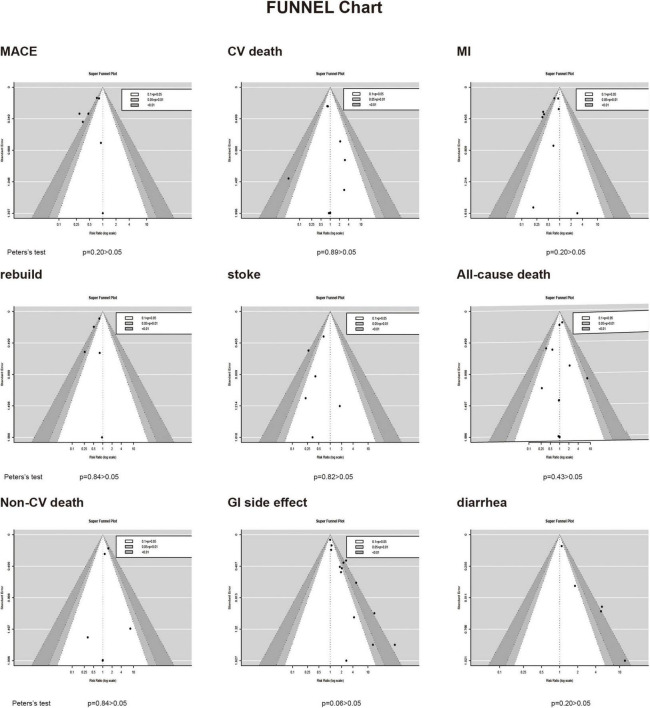
The detection of publication bias. Funnel chart.

**FIGURE 7:**
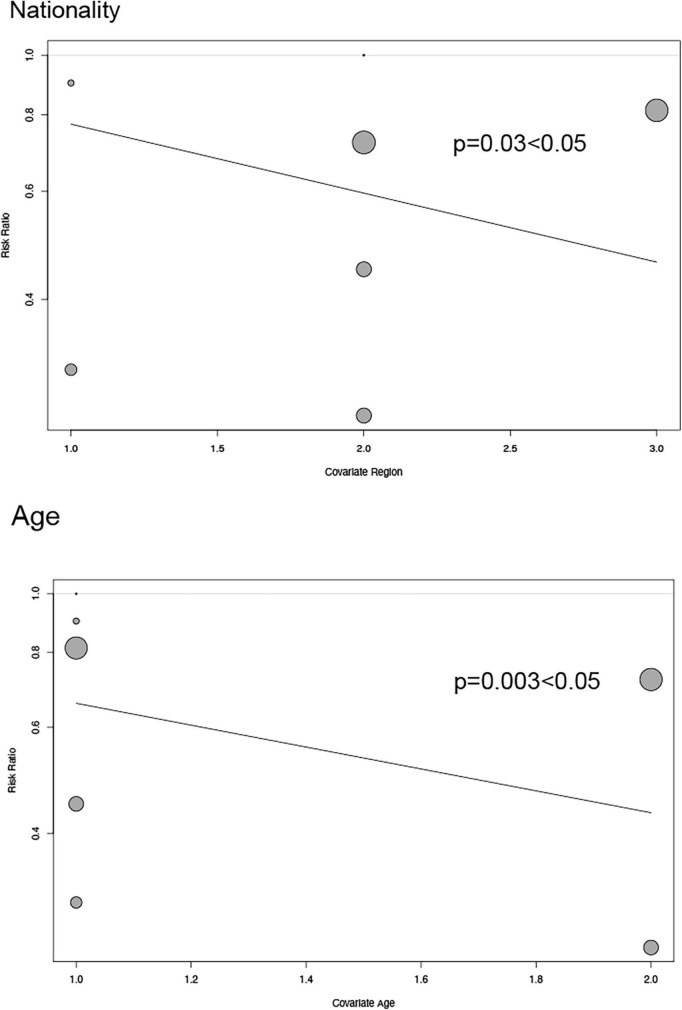
Regression plot exhibiting association between age and nationality of patients and major adverse cardiovascular events.

Depending on the heterogeneity of the included RCT, we used either a random effects model or a fixed effects model for data analysis, and we used the Peters test and funnel plot for testing the risk of bias at *p* < 0.05, which was symmetrical from the point of view of the geometry in Figure 6. This indicates that the risk of bias was low for the RCT included in our meta-analysis. we also performed a meta-regression analysis to determine outcome-related variables (Figure 7).

### Subgroup analysis

#### Duration of follow-up visits

**FIGURE 8:**
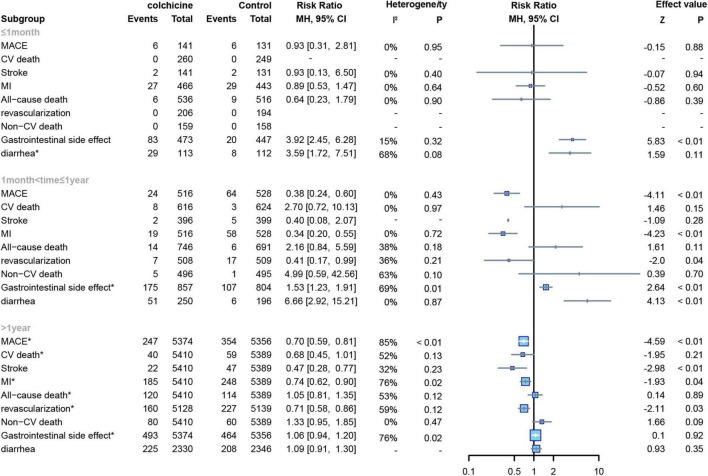
Forest plot of subgroup of study with different follow-up duration. MACE, major adverse cardiovascular events; CV death, cardiovascular death; MI, myocardial infarction; Non-CV death, Non-cardiovascular death. *Random-effect model.

Among the included studies, 7 studies had a follow-up time of ≤ 1 month (15–21), 5 studies had a follow-up time of > 1 month and ≤ 1 year (12, 22–25), and 3 studies had a follow-up time of > 1 year (7, 8, 13). First, regardless of the length of follow-up, we found no significant effect of colchicine on cardiovascular mortality, non-cardiovascular mortality, and all-cause mortality compared to controls. Secondly, we found that colchicine treatment significantly increased the incidence of gastrointestinal adverse events compared to the control group when the follow-up period was < 1 year but had no significant effect on the incidence of gastrointestinal adverse events compared to the control group when the follow-up period was > 1 year (RR: 1.06; 95% CI: 0.94–1.20; *p* for heterogeneity *p* = 0.01; I2 = 78%; *p* = 0.55); In addition, when follow-up was ≤ 1 month, the colchicine treatment group had no significant effect on cardiovascular outcomes, all-cause mortality, and non-cardiovascular mortality, whereas when follow-up was > 1 month in the study, colchicine administration reduced the risk of MACE, MI, stroke, revascularization, and non-cardiovascular mortality (Figure 8).

#### Dose of colchicine

**FIGURE 9:**
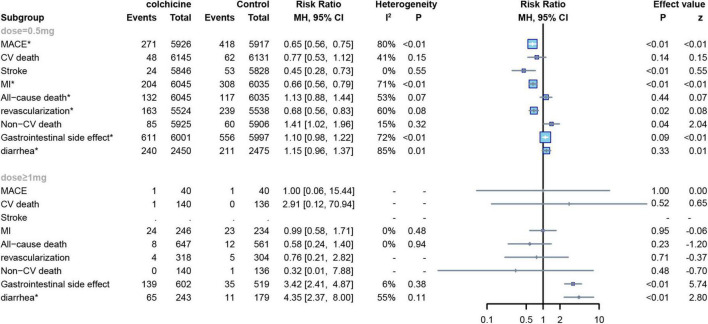
Forest plot of subgroup of the application dose of colchicine. MACE, major adverse cardiovascular events; CV death, cardiovascular death; MI, myocardial infarction; Non-CV death, Non-cardiovascular death. *Random-effect model.

Six RCTs applied low doses of colchicine (dose = 0.5 mg) to 13,165 subjects (7, 8, 12, 13, 22, 23),and eight other RCTs applied high doses of colchicine (dose ≥ 1 mg) to 1,295 subjects (7, 15–18, 20, 24, 25), One trial did not report a definitive dose (21). In our study, a subgroup analysis was conducted to establish the relationship between colchicine and cardiovascular risk. No significant differences were demonstrated in CV mortality and all-cause mortality compared to controls for either low or high dose colchicine; low dose colchicine significantly reduced the risk of MACE (RR: 0.65; 95% CI: 0.56–0.75; p for heterogeneity *p* < 0.01; I2 = 80%; *p* < 0.01), MI (RR: 0.66;95%CI:0.56–0.79;p for heterogeneity *p* < 0.01; I2 = 71%; *p* < 0.01), stroke (RR:0.45;95%CI:0.28–0.73;p for heterogeneity *p* = 0.55; I2 = 0%; *p* < 0.01), and revascularization in subjects (RR:0.68;95%CI:0.56–0.83;p for heterogeneity *p* = 0.08; I2 = 60%; *p* = 0.02). However, high-dose colchicine did not show similar benefits for MACE, MI, stroke, or revascularization. Finally, both high and low doses of colchicine increased the risk of gastrointestinal events compared to the control group, but low-dose colchicine treatment was not significantly associated with the occurrence of diarrhea (RR: 1.15; 95% CI: 0.96–1.37; p for heterogeneity *p* = 0.02; I2 = 84%; *p* = 0.33) (Figure 9).

#### Age

**FIGURE 10:**
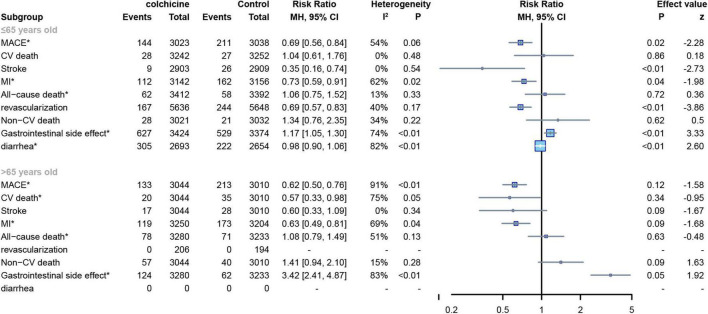
Forest plot of subgroup of subjects with varied age. MACE, major adverse cardiovascular events; CV death, cardiovascular death; MI, myocardial infarction; Non-CV death, Non-cardiovascular death. *Random-effect model.

The mean age of subjects in 11 RCTs was ≤ 65 years (8, 12, 15, 17, 19–22, 24–26) and the age of subjects in the other 4 RCTs was > 65 years (7, 13, 16, 18),For all-cause deaths, CV deaths and non-CV deaths, colchicine had no effect at all age groups, In contrast, colchicine treatment significantly reduced the risk of MACE(RR: 0.69; 95% CI: 0.56–0.84; p for heterogeneity *p* = 0.06; I2 = 54%; *p* = 0.02), stroke(RR: 0.35; 95% CI: 0.16–0.74; p for heterogeneity *p* = 0.54; I2 = 0%; *p* < 0.01), MI(RR: 0.73; 95% CI: 0.59–0.91; p for heterogeneity *p* = 0.03; I2 = 62%; *p* = 0.03) and revascularization(RR: 0.69; 95% CI: 0.57–0.83; p for heterogeneity *p* = 0.17; I2 = 40%; *p* < 0.01) in subjects up to 65 years of age, but did not show significant differences in subjects over 65 years of age (Figure 10).

#### Dosing before percutaneous coronary intervention

**FIGURE 11:**
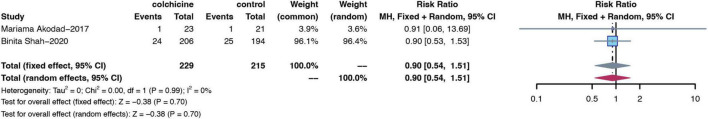
Forest plot of subgroups of MACE from studies with pre-surgical colchicine treatment. MACE, major adverse cardiovascular events.

Two RCTs (15, 16) reported the number of MACE events in patients after pre-PCI dosing. Pre-PCI colchicine did not have a significant effect on improving the risk of cardiovascular MACE in patients compared to the control group (RR: 0.90; 95% CI: 0.54–1.51; p for heterogeneity *p* = 0.99; I2 = 0%; *p* = 0.70) (Figure 11).

#### Related comorbidities

**FIGURE 12:**
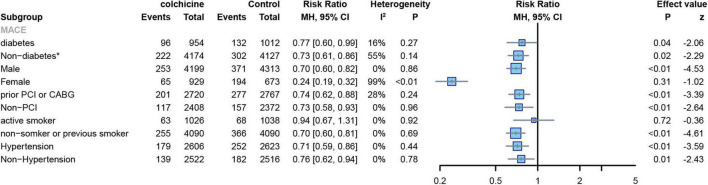
Forest plot of subgroup of the diabetes or not, sex, prior PCI or not, hypertension or not, and frequency of smoking. MACE, major adverse cardiovascular events. *Random-effect model.

Two RCTs reported the number of MACE events in patients with diabetes and hypertension (7, 8) colchicine reduces the risk of MACE in patients with or without diabetes or hypertension. (diabetes: RR: 0.77; 95% CI: 0.60–0.99; p heterogeneity *p* = 0.27; I2 = 16%; *p* = 0.03; non-diabetes: RR: 0.73; 95% CI: 0.61–0.86; p heterogeneity *p* = 0.14; I2 = 55%; *p* = 0.02;hypertension: RR: 0.71; 95% CI: 0.59–0.86; p heterogeneity *p* = 0.44; I2 = 0%; *p* < 0.01; non-hypertension: RR: 0.76; 95% CI: 0.62–0.94; p heterogeneity *p* = 0.78; I2 = 0%; *p* = 0.01) (Figure 12). Two RCTs reported the number of MACE events in patients with previous PCI or CABG (7, 8).,Patients with a reduced risk of MACE after colchicine compared to controls, regardless of whether PCI or CABG had been performed previously(prior PCI or GABG: RR: 0.74; 95% CI: 0.62–0.88; p heterogeneity *p* = 0.24; I2 = 28%; *p* < 0.01; non-PCI or GABG: RR: 0.73; 95% CI: 0.58–0.93; p heterogeneity *p* = 0.96; I2 = 0%; *p* < 0.01) (Figure 12).

#### Sex and smoking

Two RCTs reported the number of MACE events in male and female subjects (7, 8). The results showed that colchicine treatment significantly reduced the risk of MACE in men (RR: 0.70; 95% CI: 0.60–0.82; p heterogeneity *p* = 0.86; I2 = 0%; *p* < 0.01), but had no significant effect on the risk of events in women (RR: 0.24; 95% CI: 0.19–0.32; p heterogeneity *p* < 0.01; I2 = 99%; *p* = 0.31) (Figure 12). Two RCTs reported the number of MACE events in smoking and non-smoking subjects (7, 8),Colchicine significantly reduced the risk of MACE in non-smoking subjects compared with controls (RR: 0.70; 95% CI: 0.60–0.81; p for heterogeneity *p* = 0.69; I2 = 0%; *p* < 0.01), but had no significant effect on the risk of events in smoking subjects(RR:0.94; 95% CI:0.67–1.31; p for heterogeneity *p* = 0.92; I2 = 0%; *p* = 0.72) (Figure 12).

## Discussion

This study aimed to analyze the effect of colchicine on cardiovascular risk by pooling available clinical trials. The study indicates that when compared with the control group (with or without placebo), the colchicine treatment group reduced the risk of MACE, MI, non-fatal stroke, and revascularization in patients with coronary artery disease; however, it did not reduce the risk of all-cause death, cardiovascular death, or non-cardiovascular death in patients with coronary artery disease. Long-term low-dose Colchicine significantly increases cardiovascular benefits in patients with coronary artery disease compared to high-dose Colchicine, interestingly, colchicine reduced cardiovascular risk in patients under the age of 65, but there was no significant correlation in patients over the age of 65. At the same time, when given before surgery to a group of patients with PCI, colchicine did not have any long-term effects.

We showed that the colchicine treatment group had a lower risk of MACE, MI, non-fatal stroke, and revascularization than the control group (with or without placebo). However, colchicine did not improve all-cause mortality or cardiovascular mortality in patients with coronary artery disease, nor did it reduce the risk of non-cardiovascular mortality in patients, as previous meta-analyses had found (10). However, The non-cardiovascular death rate was higher in the colchicine group compared to the control group in two large RCT (*n* = 5,522; *n* = 4,745) (7, 8). We were surprised to discover that colchicine may increase the risk of non-cardiovascular mortality when we excluded the Tong DC-2020 (*n* = 795) study from the meta-analysis(RR:1.42; 95% CI:1.01–1.98; p for heterogeneity; *p* = 0.44; I2 = 0%; *p* = 0.04) (22). Therefore, we think that colchicine’s effects on non-cardiovascular mortality should be interpreted with care. To verify this reliability, more substantial RCTs are required.

At the same, subgroup analysis by colchicine dose showed that lower doses of colchicine reduced the risk of cardiovascular outcomes (MACE, MI, stroke, revascularization), whereas higher doses did not show a significant advantage in reducing the risks of cardiovascular outcomes. In a meta-analysis conducted by Thomas et al., a subgroup analysis was performed according to the colchicine dose used in each of the included studies, reporting outcomes including all-cause death, cardiovascular death, stroke/TIA, MI, and ischemia-driven revascularization, contradicting our findings in that they concluded that there was no significant difference between high and low doses regarding the incidence of stroke/TIA and ischemia-driven revascularization (27). This could be because we have clearly defined stroke as a non-fatal stroke, with both deaths from stroke and non-fatal stroke included in their outcome indicators. At the same time, we found that colchicine increased the risk of gastrointestinal symptoms at both high and low doses, but the effect was less at low doses than at high doses (high dose RR: 1.10, low dose RR: 3.42), confirming previous findings (14, 28, 29).

In addition, we discovered that differences in the duration of study follow-up contributed to differences in outcomes, as colchicine is frequently used as a lifelong treatment for chronic disease and the duration of treatment affects long-term benefits and harms. A meta-analysis by Xia et al. showed that colchicine treatment with a follow-up of more than 6 months significantly reduced the incidence of major adverse cardiovascular events in patients with coronary artery disease (30). Which is consistent with our results, but they did not specify the efficacy of short-term colchicine treatment; Tien et al. previously reported an association between colchicine and the incidence of treatment-time MI in patients with coronary artery disease after PCI (31), but some subjects who had not undergone PCI were excluded from their study, which may have led to some bias. Their meta-analysis revealed that short-term (less than 6 months) treatment with colchicine significantly reduced the risk of MI after PCI compared to long-term treatment. To reduce bias, we included all patients with acute and chronic CAD in our study and divided the follow-up into three subgroups: 1 month, > 1 month and 1 year, and 1 year. In studies with less than 1 month of follow-up, we discovered that colchicine was not associated with a benefit in cardiovascular outcomes; however, in studies with longer follow-up, colchicine produced a more notable cardiovascular benefit when compared to controls. Gastrointestinal adverse events are colchicine’s most common side effects (32). We found that the effects of colchicine on gastrointestinal side effects were less noticeable with longer follow-up. This could also be because the COLCOT (8) and LoDoCo2 trials (7), which had large sample sizes, were included in the subgroup of units that were followed for more than a year. As a result, more data are required to determine the risk of adverse gastrointestinal events over the duration of the study’s follow-up. In addition, colchicine did not affect improving all-cause and cardiovascular mortality in coronary artery disease patients, regardless of dose or duration of follow-up.

Inflammation is a major factor in atherosclerosis (33, 34). In theory, reducing inflammation levels could be a therapeutic option to reduce cardiovascular risk in patients with CAD. Bytyçi et al. recently conducted a meta-analysis that found that giving colchicine for 24 h reduced inflammatory markers (hs-CRP, IL-1, IL-6, and IL-18) in patients with unstable CAD (9). Meanwhile, the COPE-PCI trial by Cole et al. showed that when colchicine was given before PCI, it was associated with a reduction in perioperative myocardial injury and lower levels of pre-PCI Inflammation (35). Colchicine’s anti-inflammatory effect in CAD patients is well known. Surprisingly, we conducted a subgroup analysis of MACE events in subjects who had received preoperative colchicine for PCI and discovered that preoperative colchicine treatment did not affect the incidence of distant MACE events in patients. As far as we know, this study is the first to analyze the efficacy of colchicine in patients after preoperative administration of PCI. Unfortunately, due to the limited number of articles and the lack of substantial data to make definitive recommendations, this conclusion should be considered exploratory and further future studies are needed to demonstrate the long-term cardiovascular benefits of colchicine in patients after PCI.

When the included RCT studies were categorized by age, we discovered that colchicine had no correlation with age for all-cause, cardiovascular, and non-cardiovascular mortality. Colchicine produced a greater cardiovascular benefit in subjects up to 65 years of age compared to controls, whereas no significant differences were observed in subjects over 65 years of age. In the CARDIA study, Lloyd-Jones et al. (36) demonstrated that the occurrence of cardiovascular disease is closely related to a person’s age group. The over-65 age group is at higher risk than other age groups, with approximately 60–80% of people facing subclinical cardiovascular disease, this may explain the result, in subjects of the aging population, the cardiovascular benefits of colchicine were outweighed by risk factors associated with their age.

This study encounters some limitations. (1)The majority of the RCTs included in this study were conducted in Western countries, and there was a lack of data on Asians, which may have resulted in bias. (2)Although we included as many RCTs that met the inclusion criteria as possible, the number of studies included in some subgroup analyses was relatively small. More research is still required to support our results. (3)When performing subgroup analyses for age, we chose the mean age of the study for analysis, which may have been biased. (4)There was considerable heterogeneity in comparing some outcome indicators (MI, MACE) and we tried to eliminate this heterogeneity by sensitivity analysis and subgroup analysis. (5) Differences in sample size between large and small trials may affect our results. The previously mentioned limitations require more large-scale RCTs to investigate further.

## Conclusion

In this study, a meta-analysis of 15 RCTs was conducted to explore the clinical benefits and safety of colchicine after its treatment for coronary heart disease. We found that colchicine treatment reduced the risk of MACE, MI, stroke, and revascularization but had no significant effect on all-cause mortality, cardiovascular, or non-cardiovascular mortality. In addition, colchicine may increase the risk of gastrointestinal adverse effects, and long-term low-dose colchicine treatment may reduce the incidence of cardiovascular events compared to higher doses, but colchicine does not appear to reduce cardiovascular risk in patients over 65 years of age or preoperative PCI, which needs to be evaluated and explored in more large sample RCTs.

## Data availability statement

The original contributions presented in this study are included in the article/Supplementary material, further inquiries can be directed to the corresponding author/s.

## Author contributions

ZM and JC conceptualized the study and performed screening, data extraction, and data analysis by R software. KJ assessed the risk of bias. ZM, JC, and KJ performed original draft preparation, reviewing, and editing. XC supervised and funded the work. All authors contributed to the article, approved, read, and agreed to the submitted version of the manuscript.

## Footnotes

1
https://www.crd.york.ac.uk/prospero/
