# Supplementary material for: Colchicine and coronary heart disease risks: A meta-analysis of randomized controlled clinical trials
Source: Front Cardiovasc Med. 2022 Sep 12;9:947959. doi: 10.3389/fcvm.2022.947959 (PMC9512890; doi:10.3389/fcvm.2022.947959)
Supplement: Supplementary file 1 [file Data_Sheet_1.docx]

**Detailed database search strategies**

**Pubmed**

((("Coronary Artery Disease"[Mesh]) OR (((((((((((((Cardiovascular Diseases[Title/Abstract]) OR (ST-segment elevation myocardial infarction[Title/Abstract])) OR (Non-ST Elevated Myocardial Infarction[Title/Abstract])) OR (Atherosclerosi[Title/Abstract])) OR (Coronary Disease[Title/Abstract])) OR (Unstable angina[Title/Abstract])) OR (Stable CAD[Title/Abstract])) OR (Coronary Heart Disease[Title/Abstract])) OR (Chronic coronary syndrome[Title/Abstract])) OR (Myocardial Infarction[Title/Abstract])) OR (Cardiovascular Stroke[Title/Abstract])) OR (Myocardial Infarct[Title/Abstract])) OR (Heart Attack[Title/Abstract]))) OR (("Percutaneous Coronary Intervention"[Mesh]) OR (((percutaneous intervention[Title/Abstract]) OR (Percutaneous Coronary Revascularization[Title/Abstract])) OR (PCI[Title/Abstract])))) AND (("Colchicine"[Mesh]) OR ((Colchicine, (R)-Isomer[Title/Abstract]) OR (Colchicine, (+-)-Isomer[Title/Abstract])))

**Embase**

| No. | Query | Results |
| --- | --- | --- |
| #7 | #5 AND #6 | 185 |
| #6 | randomized controlled trial':ab,ti OR 'randomized':ab,ti OR 'placebo':ab,ti | 1055681 |
| #5 | #3 AND #4 | 811 |
| #4 | colchicine' | 41302 |
| #3 | #1 OR #2 | 619906 |
| #2 | percutaneous coronary intervention':ab,ti OR 'percutaneous intervention':ab,ti OR 'percutaneous coronary revascularization':ab,ti OR 'pci':ab,ti | 93475 |
| #1 | coronary artery disease':ab,ti OR 'cardiovascular diseases':ab,ti OR 'st-segment elevation myocardial infarction':ab,ti OR 'non-st elevated myocardial infarction':ab,ti OR 'atherosclerosi':ab,ti OR 'coronary disease':ab,ti OR 'unstable angina':ab,ti OR 'stable cad':ab,ti OR 'coronary heart disease':ab,ti OR 'chronic coronary syndrome':ab,ti OR 'myocardial infarction':ab,ti OR 'cardiovascular stroke':ab,ti OR 'myocardial infarct':ab,ti OR 'heart attack':ab,ti | 580553 |

**Cochrane**

#1 MeSH descriptor: [Coronary Artery Disease] explode all trees

#2 (Cardiovascular Diseases):ti,ab,kw OR (ST-segment elevation myocardial infarction):ti,ab,kw OR (Non-ST Elevated Myocardial Infarction):ti,ab,kw OR (Atherosclerosi):ti,ab,kw OR (Unstable angina):ti,ab,kw OR (Coronary Disease):ti,ab,kw OR (Coronary Heart Disease):ti,ab,kw OR (Stable CAD):ti,ab,kw OR (Chronic coronary syndrome):ti,ab,kw OR (Myocardial Infarction):ti,ab,kw OR (Myocardial Infarct):ti,ab,kw OR (Cardiovascular Stroke):ti,ab,kw OR (Heart Attack):ti,ab,kw

#3 #1 or #2

#4 (Percutaneous Coronary Intervention):ti,ab,kw OR (PCI):ti,ab,kw OR (Percutaneous Coronary Revascularization):ti,ab,kw OR (percutaneous intervention):ti,ab,kw

#5 #4 or #3

#6 Colchicine:ti,ab,kw

#7 #5 and #6

**clinicaltrial.gov**

condition or disease: Coronary heart disease

intervention/treatment:colchicine
